# Clinical outcomes of proximal gastrectomy for gastric cancer: A comparison between the double-flap technique and jejunal interposition

**DOI:** 10.1371/journal.pone.0247636

**Published:** 2021-02-24

**Authors:** Tsutomu Kumamoto, Mitsuru Sasako, Yoshinori Ishida, Yasunori Kurahashi, Hisashi Shinohara

**Affiliations:** 1 Department of Gastroenterological Surgery, Hyogo College of Medicine, Nishinomiya, Hyogo, Japan; 2 Department of Surgery, Yodogawa Christian Hospital, Higashiyodogawa, Osaka, Japan; University Hospital Hamburg Eppendorf, GERMANY

## Abstract

**Background:**

The optimal reconstruction method after proximal gastrectomy (PG) has been debatable. Recent reports have shown that the double-flap technique (DFT) provides good outcomes in terms of postoperative nutritional status and quality of life. However, no study has compared the clinical outcomes of the DFT with other reconstruction methods. Here, we evaluated and compared the clinical outcomes between the DFT and jejunal interposition (JI) after PG for gastric cancer.

**Materials and methods:**

The medical records of 34 consecutive patients who had undergone PG for upper third gastric cancer between January 2011 and October 2016 were reviewed retrospectively. The main factors investigated were surgical outcomes, postoperative nutritional status, symptoms, and endoscopic findings 1 year after surgery.

**Results:**

Thirty-four patients were enrolled (DFT, 14; JI, 20). The operation time was similar between the two techniques (228 and 246 minutes for DFT and JI, respectively, *P* = 0.377), as were the rates of anastomotic complications (7% and 0% for DFT and JI, respectively, *P* = 0.412). Body weight loss was significantly lower in the DFT group than in the JI group (-8.1% vs -16.1%, *P* = 0.001). Total protein and albumin levels were higher in the DFT group than in the JI group (0% vs -2.9%, *P* = 0.053, and -0.3% vs -6.1%, *P* = 0.077, respectively). One patient in the DFT group and no patients in the JI group experienced reflux esophagitis (≥ grade B) (*P* = 0.393). Anastomotic strictures were not observed as postoperative complications in either group.

**Conclusions:**

Surgical outcomes revealed that the DFT was safe and feasible, similar to JI. In terms of controlling postoperative body weight loss, the DFT is a better reconstruction technique than JI after PG.

## Introduction

Postoperative body weight loss always occurs in patients undergoing gastrectomy for gastric cancer, and the significant decrease in body weight and sarcopenia after gastrectomy are negatively associated with long-term survival [1, 2]. Therefore, considerable attention must be paid to maintaining the body weight and nutritional status after gastrectomy. Consequently, proximal gastrectomy (PG) is in the limelight; it is a function-preserving procedure recommended for cT1N0 gastric cancer in the upper third of the stomach in accordance with the Japanese Gastric Cancer Guidelines [3]. Recent studies have reported that PG with lymph node dissection was also sufficient as an oncological outcome for T2/T3 proximal gastric cancer [4, 5].

However, some surgeons remain reluctant to perform PG, even for early gastric cancer in the upper third of the stomach. This is because simple esophagogastrostomy after PG, which was first described in 1898 [6], is closely associated with reflux esophagitis, which has been the major critical point as it often leads to a decreased dietary intake and a deterioration in the quality of life (QOL) [7]. To overcome this problem, several reconstruction methods such as jejunal interposition (JI) and double-tract reconstruction have been devised and used after PG. Lately, the double-flap technique (DFT), devised in 1998, is an improved esophagogastrostomy technique that prevents esophageal reflux [8]. The details of this technique were more recently described in English in 2016 [9].

Recently, some studies have reported that PG is superior to total gastrectomy in terms of nutritional status including body weight loss and postoperative QOL [10–13]. However, there were a limited number of studies that compared two or more reconstruction methods after PG [12, 13] and no studies have investigated the differences in clinical outcomes between the DFT and other reconstruction methods after PG. Therefore, the optimal reconstruction method after PG remains debatable.

This study aimed to retrospectively evaluate and compare the surgical outcomes, changes in nutritional status, postoperative endoscopic findings, postoperative QOL, and late postoperative complications between the DFT and JI after PG for gastric cancer of the upper third of the stomach.

## Materials and methods

### Patients

**Fig 1:**
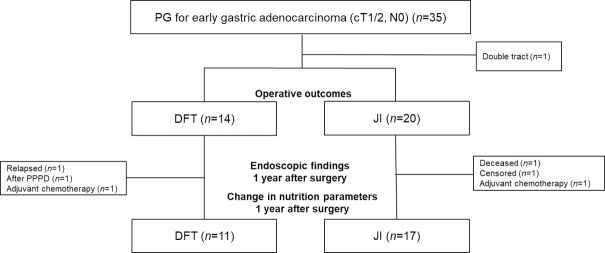
Study flowchart. Thirty-four patients were enrolled in this study, and the surgical outcomes were compared between the double-flap technique (DFT) and jejunal interposition (JI). Changes in nutritional status and endoscopic findings were evaluated for 28 patients. PPPD, pylorus-preserving pancreatoduodenectomy.

From January 2011 to October 2016, 770 patients diagnosed with gastric cancer underwent gastric cancer surgery at the Hyogo College of Medicine, Hyogo, Japan. For many years, PG was performed for patients with cT1N0 gastric cancer in the upper third of the stomach, especially for patients in whom two-thirds of the stomach could be preserved by preoperative estimation. Since 2013, the adaptation of PG has gradually expanded for patients clinically diagnosed with T2 gastric cancer. In the study period, 35 consecutive patients with primary cT1N0 or cT2N0 gastric cancer of the upper third of the stomach underwent PG at our institution. From 2011 to 2013, JI was used for reconstruction after PG in 20 patients. Since 2014, the DFT started to be performed for reconstruction after PG in the department and 14 patients underwent the DFT except for one who underwent double-tract reconstruction. Subsequently, six patients were excluded from the evaluation of post-surgical conditions including nutritional indicators, endoscopic findings, and symptoms, because of several reasons shown in Fig 1.

Preoperatively, patients were evaluated by gastrointestinal endoscopy, upper gastrointestinal radiographs, multidetector-row computed tomography, or endoscopic ultrasonography. All patients were diagnosed with adenocarcinoma by biopsy. The clinical stage was determined according to the Japanese Classification of Gastric Carcinoma, 3rd English edition [14].

This study was approved by the Ethics Committee of Hyogo College of Medicine (Institutional Review Board number 3538). The Committee waived the requirement for informed consent for this study.

### Surgical PG procedure

All operations were performed under the supervision of an experienced gastric cancer surgeon (M.S.) using an open approach. The extent of the lymph node dissections was D1+ (nodes no. 1, 2, 3a, 4sa, 4sb, 7, 8a, 9, and 11p) or more according to the Japanese Gastric Cancer Treatment Guidelines, version 5 [3]. The hepatic branch and peripheral pyloric branches of the vagus nerve were always preserved, and the celiac branch was principally preserved. The finger bougie [15] method was principally performed as a pyloric drainage procedure.

#### Reconstruction using the DFT

**Fig 2:**
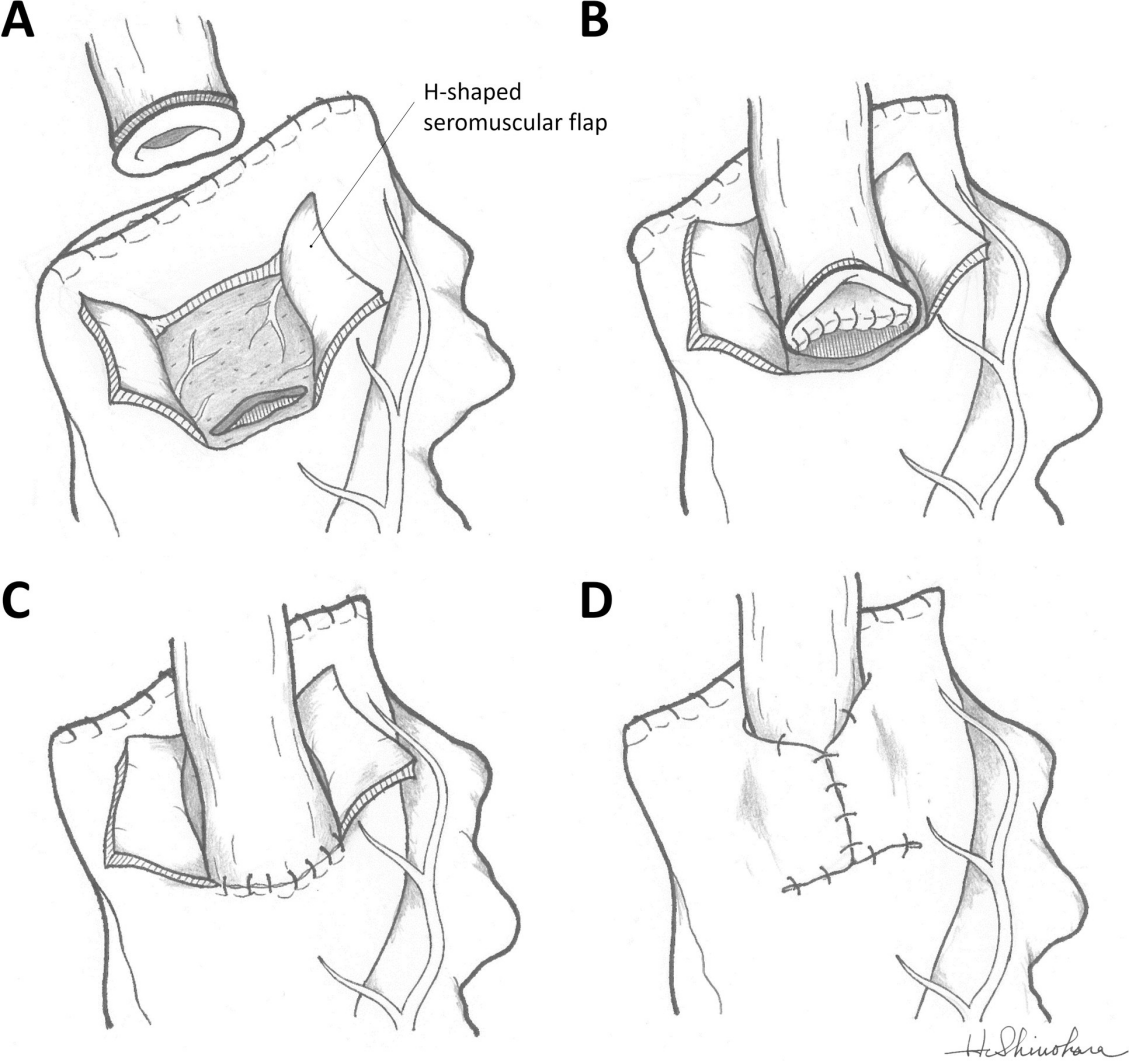
Schematic of the double-flap technique (DFT) surgical procedure. (A) An H-shaped seromuscular flap (2.5 × 3.5 cm) was created at the anterior wall of the gastric remnant. (B) After the posterior wall of the esophagus was fixed to the upper edge of the flap, the anastomosis of the posterior wall was sutured. (C) Anastomosis of the anterior wall was sutured continuously with Gambee stitches. (D) The anastomosis was covered with a Y-shape suturing of the flaps, and a valvuloplastic esophagogastrostomy using DFT was completed.

The detailed surgical procedure using the DFT has been described in previous reports [9, 16]. Firstly, an H-shaped seromuscular flap was created at the anterior wall of the gastric remnant. The width and height of the flap were 2.5 and 3.5 cm, respectively, and the top of the flap was located 3 to 4 cm from the top of the gastric remnant (Fig 2A). After the flap was created, the posterior wall of the esophagus was fixed to the upper edge of the flap by suturing at four points. Then the gastric mucosa and submucosa were opened 5 mm above the lower edge of the flap. The opened length was the same or a little larger than the width of the esophagus. Anastomosis of the posterior wall was performed by interrupted sutures that closed all layers of the esophagus to the mucosa and submucosa of the gastric remnant (Fig 2B). Anastomosis of the anterior wall was sutured by Gambee stitching (Fig 2C). Finally, the anastomosis was covered with a Y-shape suturing of the flaps using interrupted sutures (Fig 2D).

#### Reconstruction using JI

**Fig 3:**
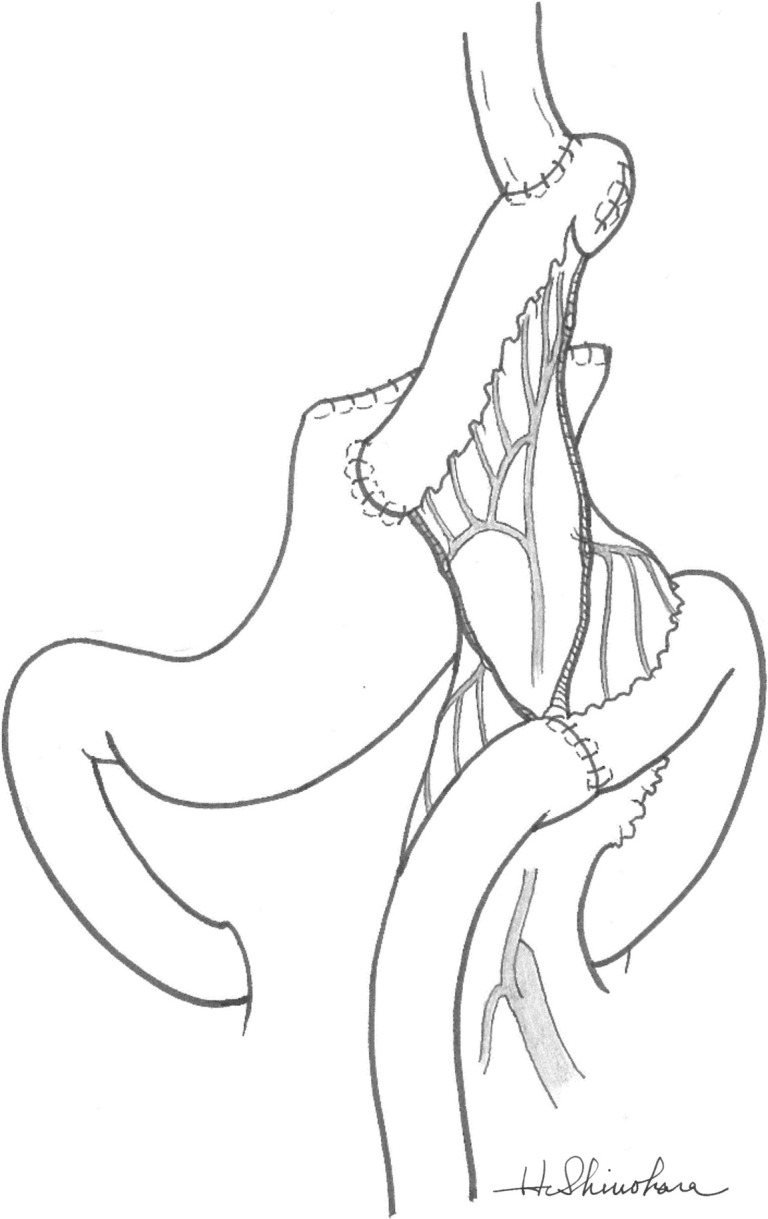
Schematic of jejunal interposition. A 10 to 13 cm jejunal limb was raised up through the retrocolic route. An end-to-side esophagojejunostomy was made using a circular stapler and an end-to-side jejunogastrostomy was made in the anterior face of the remnant stomach with Gambee stitches.

Firstly, the jejunum was divided 25–30 cm distal to the ligament of Treitz, and then 5 cm or more of the jejunum was sacrificed to raise up a 10 to 13 cm jejunal limb through the retrocolic route without cutting the distal arcade vessels of the distal mesentery. An end-to-side esophagojejunostomy was made using a 25 mm circular stapler (EEA™; Covidien, Mansfield, MA, USA), and the jejunal stump was closed using a 55-mm linear stapler (Ethicon Endo-Surgery, Cincinnati, OH, USA) and covered by seromuscular sutures to make this stump as short as 1–2 cm. An end-to-side jejunogastrostomy was made in the anterior face of the remnant stomach about 10 cm below the top by Gambee stitches. Finally, an end-to-end jejunojejunostomy was performed by Gambee stitches (Fig 3).

### Clinical analysis and surgical outcomes

The patients’ basic characteristics (age, sex, body mass index, and American Society of Anesthesiologists physical status [ASA-PS]), history of preoperative endoscopic submucosal dissection, tumor status (clinical T stage, pathological T stage, pathological N stage, and pathological stage), and adjuvant chemotherapy (yes or no) were collected from the medical records. Surgical data (operation time, estimated blood loss, combined cholecystectomy, and operative findings) and postoperative outcomes (morbidity, mortality, and postoperative hospital stays) were also obtained from the medical records. Complications of grade III or higher according to the Clavien-Dindo classification within 30 days or during the postoperative stay were defined as early complications [17].

### Follow-up and postoperative nutritional status

Routine follow-up was performed every half a year till five years at the least, by blood tests and interviewing the patients regarding their symptoms. Endoscopy was performed at 1, 3, and 5 years after surgery. Either computed tomography or abdominal ultrasonography was performed every year.

For this study, body weight and serum concentrations of total protein (TP), albumin (Alb), and hemoglobin (Hb) at 6 and 12 months after surgery were collected from the medical records.

For endoscopic findings, those at approximately 1 year after surgery were collected from the medical records and confirmed by checking the original images for cases in which patients had any positive findings such as reflux esophagitis or food residue in the organs. The Los Angeles classification [18] was used for findings of reflux esophagitis and the RGB classification [19] was used to evaluate the condition of the remnant stomach.

The patients’ digestive symptoms at 6 and 12 months after surgery were evaluated by the Post-Gastrectomy Syndrome Assessment Scale-45 [20].

Postoperative complications were reviewed during all follow-up periods. Anastomotic stricture was defined as cases that required endoscopic balloon dilations. Patients excluded from these evaluations included a patient who had relapsed, a patient who underwent PG after pylorus-preserving pancreatoduodenectomy for a pancreatic neuroendocrine tumor, a patient who died of a disease other than gastric cancer, a patient who was not able to attend a follow-up consultation, and two patients who underwent adjuvant chemotherapy for one year (Fig 1). The median follow-up time was 59 (5–89) months: 41 (15–69) months for the DFT group and 59 (5–89) months for the JI group.

### Statistical analysis

The Fisher’s exact test was used for categorical variables. The Mann-Whitney U-test was used to evaluate continuous variables, which were expressed as medians and interquartile ranges. The Student’s t test was used to express the mean and standard deviations of the data, as required. Postoperatively, the rates of body weight loss and changes in TP, Alb, and Hb were compared using repeated-measures analysis of variance (adjusted for preoperative values). Results were considered to be statistically significant when *P*<0.05. All statistical analyses were performed using Statistical Package for the Social Sciences version 24 (IBM Corp., Armonk, NY, USA).

## Results

### Clinical analysis and surgical outcomes

We assessed a total of 34 patients, which included 14 and 20 patients for the DFT and JI groups, respectively. The patient and tumor characteristics are summarized in Table 1. No significant differences were observed in the background and clinical characteristics of the two groups. The clinical T2 stage ratio was 21% in the DFT group and 5% in the JI group due to change in the indication for PG (*P* = 0.283).

**Table 1:** Baseline demographics of the patients.

Variable	DFT (*n* = 14)	JI (*n* = 20)	*P* value
Age (years)	64.3±9.1	63.2±10.2	0.725
Sex (male/female)	11/3	19/1	0.283
BMI (kg/m^2^)	24.5±3.2	23.5±2.4	0.316
ASA-PS (1/2/3)	1/12/1	4/12/4	0.298
Preoperative ESD, *n* (%)	4 (29%)	6 (30%)	1
Clinical T stage			0.283
T1	11 (79%)	19 (95%)	
T2	3 (21%)	1 (5%)	
Pathological T stage^a^			
T1	10 (71%)	16 (80%)	0.315
T2	0 (0%)	2 (10%)	
T3	4 (29%)	2 (10%)	
Pathological N stage^a^			0.365
N0	12 (86%)	19 (95%)	
N1	1 (7%)	0 (0%)	
N2	0 (0%)	1 (5%)	
N3	1 (7%)	0 (0%)	
Pathological stage^a^			0.276
IA	9 (64%)	16 (80%)	
IB	4 (29%)	2 (10%)	
IIA	0 (0%)	1 (5%)	
IIIA	0 (0%)	1 (5%)	
IIIB	1 (7%)	0 (0%)	
Adjuvant chemotherapy, *n* (%)	1 (7%)	1 (5%)	1

DFT, double-flap technique; JI, jejunal interposition method; BMI, body mass index; ASA-PS, American Society of Anesthesiologists physical status; ESD, endoscopic submucosal dissection

Variables are described using mean ± standard deviations, and the *P* values were calculated using the Student’s t test.

^a^ According to the Japanese Classification of Gastric Carcinoma, 3^rd^ English edition

Surgical outcomes and early postoperative complications are shown in Table 2. No significant differences were observed in the median operation time between the DFT and JI groups (228 min vs 246 min, *P* = 0.377), but the interquartile range of the operation time was less in the JI group than in the DFT group (196–273 min vs 227–270 min). The DFT group experienced significantly lesser blood loss than the JI group (250 ml vs 435 ml, *P* = 0.015). In terms of postoperative complications (CD ≥ 3), an anastomotic leakage and a pancreatic fistula occurred only in the DFT group, but this did not significantly differ from the JI group (*P* = 0.412). Anastomotic stricture did not occur in either group. The median postoperative hospital stay was shorter for the JI group than for the DFT group (*P* = 0.018).

**Table 2:** Surgical outcomes and early postoperative complications.

Variable	DFT (*n* = 14)	JI (*n* = 20)	*P* value
Operation time (min)^a^	228 (196–273)	246 (227–270)	0.377
Estimated blood loss (ml)^a^	250 (198–318)	435 (286–573)	0.015
Combined cholecystectomy	2 (14%)	3 (15%)	1
Morbidity (CD ≥ 3)^b^	2 (14%)	0 (0%)	0.162
Anastomotic complication	1 (7%)	0 (0%)	0.412
Leakage	1 (7%)	0 (0%)	0.412
Stricture	0 (0%)	0 (0%)	1
Pancreatic fistula	1 (7%)	0 (0%)	0.412
Mortality	0 (0%)	0 (0%)	1
Postoperative hospital stay (days)^a^	12 (11–19)	11 (10–12)	0.018

DFT, double-flap technique; JI, jejunal interposition method

^a^ Variables are described using medians and interquartile ranges, and the *P* values were calculated using the Mann-Whitney U-test.

^b^ According to the Clavien-Dindo classification

### Postoperative conditions in follow-up period

#### Nutritional status

**Fig 4:**
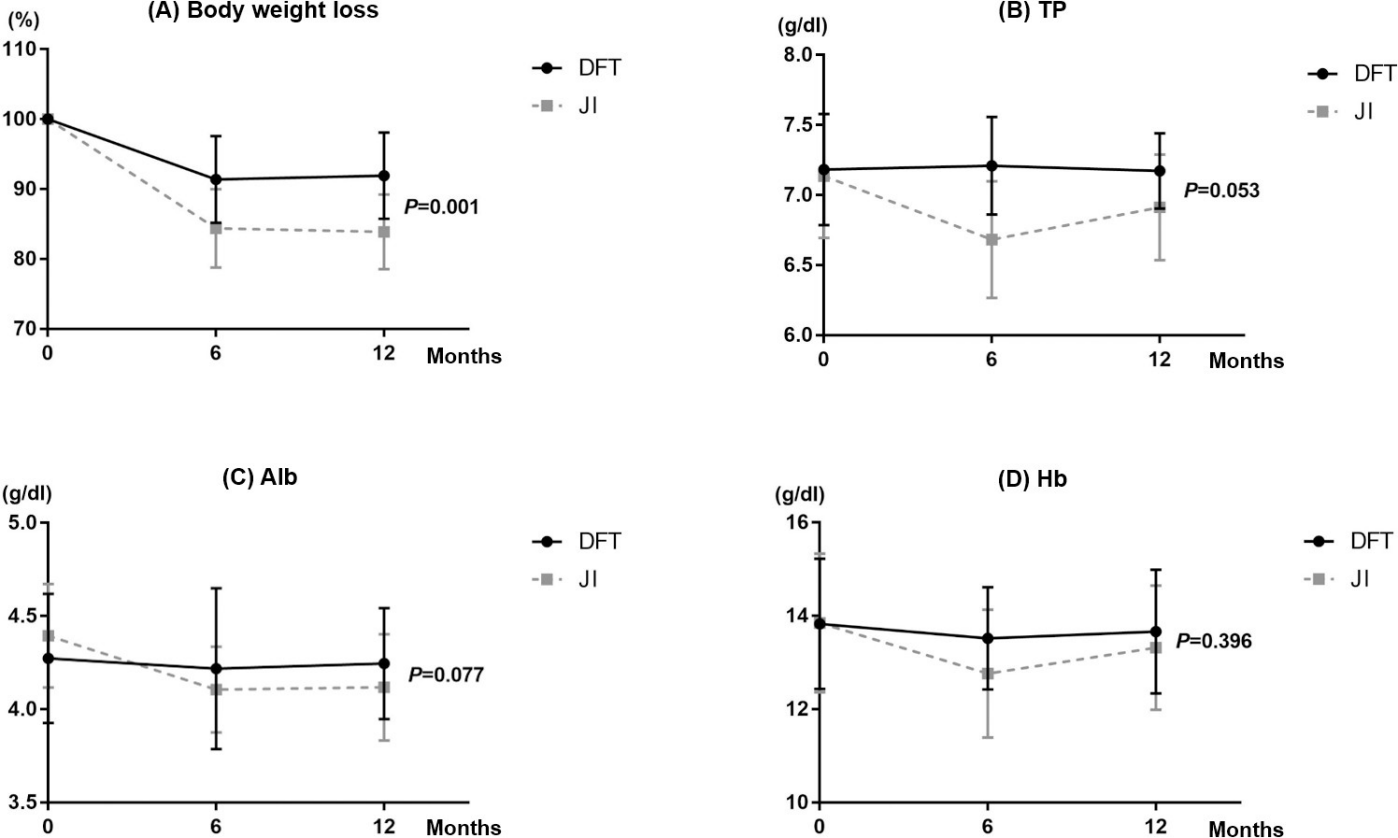
Comparison of changes in the (A) body weight loss, (B) total protein (TP), (C) albumin (Alb), and (D) hemoglobin (Hb) between the double-flap technique (DFT) and jejunal interposition (JI).

Regarding comorbidities, no significant differences were noted between the DFT and JI groups (S1 Table). The changes in pre- and postoperative nutritional status (mean body weight loss, TP, Alb, and Hb) are shown in Fig 4. The body weight loss percentage at 1 year after surgery in the DFT group was significantly lower than that in the JI group (-8.1% vs -16.1%, *P* = 0.001). Of clinical significance, the postoperative mean TP and Alb loss tended to be lower in the DFT group than in the JI group (0% vs -2.9%, *P* = 0.053 and -0.3% vs -6.1%, *P* = 0.077, respectively). The mean Hb did not significantly differ between the two groups (-0.6% vs -3.4%, *P* = 0.396).

#### Endoscopic findings

Endoscopic findings 1 year after surgery are shown in Table 3. Regarding reflux esophagitis, no significant differences were noted; however, there was one patient with grade B reflux esophagitis in the DFT group (*P* = 0.393). No significant differences were found in terms of RGB classification between the DFT and JI groups (36% vs 44%, *P* = 1.000). Esophageal residue was only observed in the JI group (0% vs 35%, *P* = 0.055). A remnant stomach was observed by gastrointestinal endoscopy in all patients.

**Table 3:** Endoscopic findings at 1 year after surgery.

	DFT (*n* = 11)	JI (*n* = 17)	*P* value
Los Angeles classification			0.393
A	0 (0%)	0 (0%)	
B	1 (9%)	0 (0%)	
C or D	0 (0%)	0 (0%)	
RGB classification			1
Residual food (≥ Grade 2)	4 (36%)	7 (41%)	
Esophageal residue and reverse	0 (0%)	6 (35%)	0.055

DFT, double-flap technique; JI, jejunal interposition method

#### Postoperative symptoms

Postoperative digestive symptoms are presented in Table 4. Overall, three (27%) and nine (53%) patients experienced digestive symptoms in the DFT and JI groups, respectively (*P* = 0.253). Of these, two (18%) and 6 (35%) patients had symptoms related to esophageal reflux or meal-related distress, respectively (*P* = 0.419).

**Table 4:** Postoperative digestive symptoms of the patients.

Variable	DFT (*n* = 11)	JI (*n* = 17)	*P* value
Total symptom	3 (27%)	9 (53%)	0.253
Esophageal reflux	1 (9%)	4 (24%)	0.619
Abdominal pain	0 (0%)	0 (0%)	1
Meal-related distress	1 (9%)	2 (12%)	1
Indigestion	0 (0%)	0 (0%)	1
Diarrhea	0 (0%)	2 (12%)	0.505
Constipation	1 (9%)	0 (0%)	0.393
Dumping	0 (0%)	1 (6%)	1

#### Late postoperative complications

During the follow-up period, anastomotic strictures and intestinal obstructions were not observed in either group. A gastric ulcer occurred in one patient in the DFT group, and an anastomotic ulcer and cholecystitis occurred in one patient in the JI group.

## Discussion

The current study investigated possible methods for reconstruction after PG for upper third cT1 or T2N0 gastric cancer that may be recommended for future surgeries. In both the DFT and JI groups, the surgical outcomes indicated that both procedures are safe and feasible. The postoperative stay was one day longer for the DFT group than for the JI group, with statistical significance but minimal clinical significance. The DFT was superior to JI in terms of postoperative nutritional status, especially for changes in the rate of body weight loss.

The operation time did not differ significantly between the groups; however, the operation time tended to be more consistent (as indicated by the interquartile range) in the JI group than in the DFT group. Moreover, only one anastomotic complication occurred in the DFT group, and none in the JI group. This complication occurred in the second case in the department. The leading surgeon (M.S.) had performed JI for more than 30 years and JI was considered technically stable and reliable before the study period [21], while the DFT started to be performed from 2014 onwards in our institution. Because the sample size of the current study is too small to evaluate the learning effect, we can regard all the cases in the DFT group under the process of learning. It is anticipated that as we perform more DFT procedures, the operation time, postoperative complications rate, and hospital stay will decrease. The surgical outcomes of the DFT in our study, however, were comparable to those in the previous reports that investigated the DFT [9, 16, 22–25]. This could be explained by the fact that all except one are reports of their initial experience with the DFT. These satisfactory outcomes in our study may be attributed to the fact that all patients underwent PG under the supervision of a well-experienced gastric cancer surgeon.

The DFT showed a significant advantage over JI in terms of postoperative nutritional status, especially in the body weight loss rate. Throughout the postoperative period, the body weights of the patients were maintained, similar to that in a previous study on the DFT [22]. We speculated that the JI group experienced greater postoperative body weight loss due to the difference in the peristaltic motion between the esophagus and interposed jejunum, which may have disturbed the passage of food at the anastomosis during the first or second postoperative years [26]. In support of this hypothesis, esophageal residue was observed only in the JI group and esophageal reflux syndrome or meal-related complaints were recognized more frequently with JI in this study, despite the lack of statistical significance. One study also indicated that JI was associated with an increase in post-gastrectomy symptoms within 30 minutes after a meal compared with esophagogastrostomy [27]. Among the patients in the DFT and JI groups, there were no significant differences in postoperative Hb levels. However, this result might be expected, because PG preserves the entire gastric antrum and not negligible part of the gastric body, which produces both gastric juice and the Castle intrinsic factor [28], both of which are related to the absorption of iron and vitamin B12. Moreover, the hepatic and pyloric branches of the vagus nerve—which can affect the function of both gastric acid and pancreatic juice excretion—were preserved in both groups in this study.

In this study, only one (9%) patient who underwent PG with the DFT was diagnosed with reflux esophagitis (≥ grade B), while anastomotic stricture was not observed in any patient. Previous studies have reported esophageal reflux (≥ grade B) and anastomotic stricture following DFT in 0–6% and 4.7–29.1%, respectively [9, 16, 22–25]. Anastomotic stricture tends to be more frequent than esophageal reflux following the DFT. Because anastomotic stricture and esophageal reflux are closely related, and meticulous procedures are required to create the valvuloplasty that prevents esophageal reflux. A study suggested that the DFT using the laparoscopic approach was the only risk factor for anastomosis-related complications [24]. However, another study in which most laparoscopic PG procedures were performed with the DFT found that the anastomosis-related complication rate was not particularly high [25]. The DFT is a technically demanding procedure; therefore, this approach must be applied carefully, based on the skill level of the surgeons.

This study had some limitations that warrant mentioning. First, this was a retrospective study that included a small sample size of patients at a single institution. Second, this was a preliminary study that focused on our initial experience with the DFT; however, good outcomes were obtained. Third, data on postoperative digestive symptoms were collected from medical records and the Post-Gastrectomy Syndrome Assessment Scale-45 questionnaire was not filled out completely. Therefore, further prospective studies are required to validate these outcomes.

## Conclusions

Surgical outcomes revealed that the DFT was safe and feasible. A priori, the DFT is recommended for reconstruction after PG to maintain postoperative nutritional status, especially in terms of controlling body weight loss. Further multicenter non-randomized studies, with larger sample sizes, which focus on reconstruction after PG are required to determine the optimal reconstruction technique after PG.

## Supporting information

S1 Table Comparison of the comorbidities in the DFT and JI groups. (DOCX)

S1 Raw data (XLSX)
