# Supplementary material for: Clinical outcomes of proximal gastrectomy for gastric cancer: A comparison between the double-flap technique and jejunal interposition
Source: PLoS One. 2021 Feb 24;16(2):e0247636. doi: 10.1371/journal.pone.0247636 (PMC7904176; doi:10.1371/journal.pone.0247636)
Supplement: S1 Table — (DOCX) [file pone.0247636.s001.docx]

S1 Table. Comparison of the comorbidities in the DFT and JI groups

| Variable | DFT (*n*=11) | JI (*n*=17) | *P* value |
| --- | --- | --- | --- |
| Comorbidities |  |  |  |
| Cardiology (angina, valve) | 2 (18%) | 1 (6%) | 0.543 |
| Pulmonary (COPD, asthma) | 1 (9%) | 1 (6%) | 1 |
| Liver disease (cirrhosis, hepatitis) | 1 (9%) | 0 (0%) | 0.393 |
| Kidney disease | 0 (0%) | 0 (0%) | 1 |
| Diabetes | 1 (9%) | 3 (18%) | 1 |
| Other malignancies | 0 (0%) | 3 (18%) | 0.258 |

COPD, chronic obstructive pulmonary disease
